# Adherence to European Society of Gastrointestinal Endoscopy Quality Performance Measures for Upper and Lower Gastrointestinal Endoscopy: A Nationwide Survey From the Italian Society of Digestive Endoscopy

**DOI:** 10.3389/fmed.2022.868449

**Published:** 2022-04-06

**Authors:** Rocco Maurizio Zagari, Leonardo Frazzoni, Lorenzo Fuccio, Helga Bertani, Stefano Francesco Crinò, Andrea Magarotto, Elton Dajti, Andrea Tringali, Paola Da Massa Carrara, Gianpaolo Cengia, Enrico Ciliberto, Rita Conigliaro, Bastianello Germanà, Antonietta Lamazza, Antonio Pisani, Giancarlo Spinzi, Maurizio Capelli, Franco Bazzoli, Luigi Pasquale

**Affiliations:** ^1^Gastroenterology Unit, IRCCS Azienda Ospedaliero-Universitaria di Bologna, Bologna, Italy; ^2^Department of Medical and Surgical Sciences, University of Bologna, Bologna, Italy; ^3^Gastroenterology and Endoscopy Unit, Azienda Ospedaliera-Universitaria Policlinico di Modena, Modena, Italy; ^4^Gastroenterology and Digestive Endoscopy Unit, Pancreas Institute, University of Verona, Verona, Italy; ^5^Diagnostic and Therapeutic Endoscopy Unit, Fondazione IRCCS Istituto Nazionale Tumori, Milan, Italy; ^6^Digestive Endoscopy Unit, Fondazione Policlinico Universitario A. Gemelli IRCCS, Università Cattolica del Sacro Cuore, Rome, Italy; ^7^Digestive Endoscopy Unit, S. Luca Hospital, Lucca, Italy; ^8^Digestive Endoscopy Unit, Manerbio Hospital, Manerbio, Italy; ^9^Gastroenterology and Digestive Endoscopy Unit, S. Giovanni di Dio Hospital, Crotone, Italy; ^10^Gastroenterology and Digestive Endoscopy Unit, Baggiovara University Hospital, Baggiovara, Italy; ^11^Gastroenterology and Digestive Endoscopy Unit, S. Martino Hospital, Belluno, Italy; ^12^Department of Surgery “Pietro Valdoni”, University La Sapienza, Rome, Italy; ^13^National Institute of Gastroenterology IRCCS Saverio de Bellis, Castellana Grotte, Bari, Italy; ^14^Gastroenterology and Endoscopy Department, Valduce Hospital, Como, Italy; ^15^Kiwa Cermet Certification Body, Statistical Department, Bologna, Italy; ^16^UOC Gastroenterologia ed Endoscopia Digestiva, Ospedale Frangipane, Avellino, Italy

**Keywords:** endoscopy, quality, performance measure, ESGE, guidelines

## Abstract

**Background:**

The quality of gastrointestinal (GI) endoscopy has been recently identified as a major priority being associated with many outcomes and patient's experience.

**Objective:**

To assess adherence of endoscopists to the European Society of Gastrointestinal Endoscopy (ESGE) quality performance measures for upper and lower GI endoscopy in Italy.

**Methods:**

All endoscopist members of the Italian Society of Digestive Endoscopy (SIED) were invited from October 2018 to December 2018 to participate to a self-administered questionnaire-based survey. The questionnaire included questions on demographics and professional characteristics, and the recent ESGE quality performance measures for upper and lower GI endoscopy.

**Results:**

A total of 392 endoscopists participated in the study. Only a minority (18.2%) of participants recorded the duration of esophagogastroduodenoscopy (EGD) and 51% provided accurate photo documentation in the minimum standard of 90% of cases. Almost all endoscopists correctly used Prague and Los Angeles classifications (87.8% and 98.2%, respectively), as well as Seattle and Management of precancerous conditions and lesions in the stomach (MAPS) biopsy protocols (86.5% and 91.4%, respectively). However, only 52.8% of participants monitored complications after therapeutic EGD, and 40.8% recorded patients with a diagnosis of Barrett's esophagus (BE). With regard to colonoscopy, almost all endoscopists (93.9%) used the Boston Bowel Preparation Scale for measuring bowel preparation quality and reported a cecal intubation rate ≥90%. However, about a quarter (26.2%) of participants reported an adenoma detection rate of <25%, only 52.8% applied an appropriate polypectomy technique, 48% monitored complications after the procedure, and 12.4% measured patient's experience.

**Conclusion:**

The adherence of endoscopists to ESGE performance measures for GI endoscopy is sub-optimal in Italy. There is a need to disseminate and implement performance measures and endorse educational and scientific interventions on the quality of endoscopy.

## Introduction

The European Society of Gastrointestinal Endoscopy (ESGE) and United European Gastroenterology (UEG) have recently identified the quality of endoscopy as a major priority (1). A quality improvement committee was instituted and performance measures were developed with the aim to improve the global quality of gastrointestinal (GI) endoscopy in Europe (2, 3). Key and minor performance measures have been identified according to various domains, which follow the track of the patient and endoscopist throughout the process of endoscopy. Quality indicators for GI endoscopy include transparency and completeness of procedure documentation, identification and management of pathology, and registration of procedural complications. Such measures have been identified following some requirements, such as proven impact on diagnostic quality and patient outcome, ease to measure and track, and susceptibility for improvement (2, 3).

Quality performance measures are related to major outcomes including detection of pre-cancerous lesions and malignancies and safety of the procedure. Esophagogastroduodenoscopy (EGD) is a widespread procedure for detection and surveillance of pre-cancerous lesions and cancers of the esophagus and stomach, and the highest quality standard is warranted (4). On the other hand, performance measures for colonoscopy, such as adenoma detection rate (ADR) and polypectomy technique, are associated with the risk of interval colorectal cancer (CRC) and mortality (5, 6). Likewise, monitoring complications after endoscopy is essential to assess the safety of procedures and identify possible targets for improvements. Meeting quality performance measures should lead to improving practice in endoscopy, patient management—both in terms of diagnostic yield and therapeutic endoscopy—and patient experience at the same time. However, such an ambitious objective might be hampered by a suboptimal adherence to quality performance measures. There is some evidence that there has been little change over time in clinical practice despite the publication of guidelines for quality in endoscopy in the US (7). There is no doubt that the implementation of quality performance measures in clinical practice might be relatively difficult, and may also depend on the availability of software for novel endoscopy reporting systems (8).

The adherence to ESGE quality performance measures for upper and lower GI endoscopy is not well-known in Europe. In fact, given the lack of quality indicators until recently, few studies assessing the quality of EGD in clinical practice have been conducted, so far. The studies providing such information are essential to disseminate and implement performance measures and improve the quality of endoscopy in Europe. In addition, such studies could inform scientific societies on the need for targeted educational interventions that may be effective in increasing knowledge and compliance with these recent quality criteria.

The aim of this survey was to assess the adherence of endoscopists to ESGE quality performance measures for upper and lower GI endoscopy in Italy.

## Materials and Methods

This survey was carried out by the Italian Society of Digestive Endoscopy (SIED) from October to December 2018. All members of SIED were eligible to participate in the study, excluding those still in training or retired. A self-administered questionnaire was distributed to the attendees of the annual SIED National Course held in Milan, Italy, in October 2018; thereafter, a link to an online version of the questionnaire was emailed to the remaining SIED members who did not attend the course. Data were collected and managed using REDCap (Research Electronic Data Capture), hosted at the Department of Medical and Surgical Sciences, University of Bologna. The REDCap is a secure web application designed to support data capture for research studies (9). Only one response *per* IP address was permitted. There were no fees or incentives for participation in the survey. The study was an initiative of the SIED Scientific Committee and was conducted after the approval by the governing council of the society itself. Written informed consent to anonymous use of data provided in the questionnaire was individually obtained from all participants.

### Questionnaire

The questionnaire was developed according to ESGE quality performance measures for upper (2) and lower (3) GI endoscopy. The SIED scientific committee selected a set of performance measures, taking into consideration local factors, such as performance measure relevance and feasibility of performance measure implementation in clinical practice. Finally, nearly all key performance measures, in particular 5 out of 6 for upper endoscopy and 6 out of 7 for lower endoscopy, were included. To reduce the number of questions and keep the survey feasible, we included only two of the minor ESGE performance measures as they were related to precancerous conditions, i.e., Barrett's esophagus and atrophic gastritis/intestinal metaplasia, of two relevant malignancies of the upper GI tract, namely, esophageal and gastric cancers. The questionnaire consisted of three sections including a total of 20 multiple-choice questions. The first section contained six questions regarding the demographic and professional characteristics of participants. The second section contained eight questions regarding five key and two minor performance measures for upper GI endoscopy. The key performance measures assessed were the following: (1) documentation of procedure duration from intubation to extubation, (2) accurate photo documentation of anatomical landmarks and all abnormal findings, (3) use of standardized disease-related terminology, (4) application of Seattle biopsy protocol in the surveillance of Barrett's esophagus (BE), and (5) registration of immediate and delayed (after 7 and 14 days) complications (bleeding, perforation) after therapeutic endoscopy (i.e., Savary/pneumatic dilation, endoscopic resection of lesions, etc.). The minor performance measures were: (1) application of validated biopsy protocol to detect atrophic gastritis and intestinal metaplasia, and (2) registration of patients with a diagnosis of BE. The third section contained six questions regarding six key performance measures for lower GI endoscopy, namely (1) bowel preparation, (2) cecal intubation rate, (3) ADR, (4) appropriate polypectomy technique, (5) registration of immediate and delayed (7-day or 30-day hospital readmission rates) complications, such as bleeding, perforation and sedation-related adverse events, after screening, diagnostic and therapeutic colonoscopy, and (6) patient's experience during and after the procedure. The questionnaire is provided in Appendix S1.

### Statistical Analysis

Continuous variables were described as mean and standard deviation (SD) if normally distributed, otherwise, as median and interquartile range. Categorical variables were described as absolute proportion and percentage. The comparison between the study sample and the SIED population was performed through the multinomial goodness-of-fit test and the *z*-test for categorical and continuous variables, respectively. Multivariable logistic regression analyses were performed to identify predictors of adherence to performance measures for upper and lower GI endoscopy. Adherence to performance measures was arbitrarily defined as a response adherent to guidelines in at least 75% of questions, which means at least 6 out of 8 (75%) questions for upper GI endoscopy and 5 out of 6 (83.3%) questions for lower GI endoscopy. Odds ratios (ORs) and 95% confidence interval (CI) were calculated after adjusting for age, gender, area of residence (North vs. Center/South), practice setting (academic vs. community/private hospital), practice duration (<10 years vs. ≥10 years), and attendance to training courses on quality of endoscopy in the previous 5 years. A *p*-value <0.05 was considered statistically significant. Statistical analysis was performed using STATA 16 (Stata Corp., College Station, Texas).

## Results

Of the 964 endoscopist members of SIED, after excluding trainees and those retired, 392 (40.7%) participated in the study. Not all participants answered all the questions; therefore, the number of responses to each question varied accordingly. Among the participants, 65.1% were men and the mean age was 49.5 years. About half of endoscopists (57.4%) practiced at community hospitals, whereas 26.3% worked in academic hospitals and 16.3% in private hospitals. Overall, 69.1% of participants practiced endoscopy for more than 10 years and most participants (82.4%) attended a training course on the quality of GI endoscopy in the last 5 years. Participants were similar to the eligible population of SIED members in terms of gender and age, but were more frequently from the North of Italy (*p* < 0.001) and academic hospitals (*p* < 0.001). Table 1 shows the demographic and professional characteristics of participants and eligible members of SIED.

**Table 1:** Demographic and professional characteristics of participants.

	**Participants,** ***n* = 392**	**Members of SIED,** ***n* = 940**	***p*-value**
	***n* (%)**	***n* (%)**	
Gender
Male	255 (65.1)	635 (67.6)	
Female	137 (34.9)	305 (32.4)	0.290
Mean age, years (SD)	49.5 (13.6)	49.6 (11)	0.897
Area of residence
North-East	74 (18.9)	164 (17.5)	
North-West	114 (29.1)	189 (20.1)	
Center	91 (23.2)	286 (30.4)	
South and Islands	113 (28.8)	301 (32)	<0.001
Practice setting
Community hospital	225 (57.4)	643 (68.4)	
Academic hospital	103 (26.3)	178 (18.9)	
Private hospital	64 (16.3)	119 (12.7)	<0.001
Practice duration (years)
<5	56 (14.3)		
5–10	65 (16.6)		
11–15	51 (13)		
16–20	53 (13.5)	-	
>20	167 (42.6)		
Attendance to training course on quality of endoscopy over the last 5 years
No	69 (17.6)		
Yes	323 (82.4)		

*SD, standard deviation*.

### Quality of Upper GI Endoscopy

A minority (18.2%) of endoscopists recorded the duration of EGD from intubation to extubation in the minimum standard of 90% of cases; similarly, only 51% provided accurate photo documentation of anatomical landmarks and all abnormal findings of the upper GI tract. Almost all participants correctly used the Prague classification for BE and the Los Angeles classification for erosive esophagitis (87.8% and 98.2%, respectively). Likewise, most endoscopists applied the Seattle biopsy protocol (86.5%) in the surveillance of BE, and the biopsy protocol according to MAPS guidelines (91.4%) in the diagnosis as well as staging of atrophic gastritis and intestinal metaplasia. Of note, only half (52.8%) of endoscopists monitored immediate and delayed complications after therapeutic EGD; in addition, less than half (40.8%) entered the patients with a diagnosis of BE into a registry and only 17% complied with the minimum standard of this performance measure (i.e., 85% of patients with BE). Table 2 shows responses regarding performance measures for upper GI endoscopy.

**Table 2:** Quality of upper gastrointestinal endoscopy.

	**Participants,** ***n* = 392**
	***n* (%)**
**Key performance measure:** Documentation of procedure duration.	
**Question:** Percentage of endoscopy reports recording the duration of the procedure^a^.	
<90%	320 (81.8)
≥90%^*^	71 (18.2)
I do not record the duration of the procedure	0
**Key performance measure:** Accurate photodocumentation.	
**Question:** Percentage of endoscopy reports with photodocumentation of anatomical landmarks and abnormal findings.	
<90%	136 (34.7)
≥90%^*^	200 (51)
I do not provide photodocumentation	56 (14.3)
**Key performance measure:** Application of standardized terminology.	
**Question:** Classification of BE.	
Simple description of length of Barrett's mucosa	29 (7.4)
Short and Long Barrett's esophagus	13 (3.3)
Prague C & M classification	344 (87.8)
None	6 (1.5)
**Question:** Classification of erosive esophagitis.	
Hetzel-Dent classification (Grade 1–4)	0
Savary-Miller classification (Grade 1–4)	5 (1.3)
Los Angeles classification (Grade A-D)	385 (98.2)
None	2 (0.5)
**Key performance measure:** Application of the Seattle protocol in BE.	
**Question:** Biopsy protocol in BE surveillance.	
1–2 random biopsies along BE	14 (3.6)
3–4 random biopsies along BE	37 (9.4)
4 biopsies taken every 2 cm along BE (Seattle protocol)	339 (86.5)
I do not take biopsies	2 (0.5)
**Key performance measure:** Monitoring complications after therapeutic endoscopy.	
**Question:** Percentage of patients monitored for complications after therapeutic endoscopy^b^.	
<95%	78 (20.7)
≥95%^*^	126 (32.1)
I do not monitor complications after therapeutic endoscopy	185 (47.2)
**Minor performance measure:** Application of biopsy protocol according to MAPS.	
**Question:** Biopsy protocol for identification of gastric atrophy and intestinal metaplasia^b^.	
1 biopsy from the antrum and 1 biopsy from the corpus	9 (2.3)
≥2 biopsies only from the antrum	6 (1.6)
≥2 biopsies from the antrum and ≥2 from the corpus (MAPS guideline)	360 (92.5)
None	14 (3.6)
**Minor performance measure:** Prospective registration of BE patients.	
**Question:** Percentage of patients with diagnosis of BE entered into a registry to monitor the incidence of dysplasia^c^.	
<85%	93 (23.9)
≥85%^*^	66 (16.9)
I do not have a registry for BE	231 (59.2)

* *Minimum standard according to ESGE performance measures*.

*BE, Barrett's esophagus*.

a *Missing data for 1 participant*.

b *Missing data for 3 participants*.

c *Missing data for 2 participants*.

### Quality of Lower GI Endoscopy

Almost all participants (95.8%) correctly used a validated scale for measuring bowel preparation quality, the Boston Bowel Preparation Scale being the preferred one (93.9%), and reported a cecal intubation rate of ≥90% (96.6%). The majority of endoscopists referred an ADR ≥25%, but 26.2% did not reach this minimum standard. Only half (52.8%) of endoscopists applied an appropriate polypectomy technique, using snare (cold or with diathermy) for polyps with >3 mm in size in the minimum standard of 80% of cases. Similarly, only 48% of endoscopists recorded immediate and delayed complications after screening, diagnostic or therapeutic colonoscopies. Most endoscopists (71.6%) did not follow ESGE recommendations to measure patient experience during and after colonoscopy using a validated questionnaire; only 12.4% of participants measured patient experience in the minimum standard of 90% of patients. Table 3 shows responses regarding performance measures for lower GI endoscopy.

**Table 3:** Quality of lower gastrointestinal endoscopy.

	**Participants,** ***n* = 392**
	***n* (%)**
**Key performance measure:** Adequate bowel preparation.	
**Question:** Scale for measuring bowel preparation quality^a^.	
Boston Bowel Preparation Scale	358 (93.9)
Ottawa Scale	3 (0.8)
Aronchick Scale	4 (1.1)
None	16 (4.2)
**Key performance measure:** Cecal intubation rate.	
**Question:** Percentage of colonoscopies reaching the cecum^a^.	
<80%	13 (3.4)
≥90%^*^	55 (12.4)
≥95%^**^	313 (82.2)
**Key performance measure:** Adenoma detection rate.	
**Question:** Percentage of colonoscopies in which at least one adenoma is identified^a^.	
<25%	100 (26.2)
≥25%^*^	281 (73.8)
**Key performance measure:** Appropriate polypectomy technique.	
**Question:** Percentage of polyps >3 mm removed with snare (cold or with diathermy) polypectomy^b^.	
<80%	178 (47.3)
≥80%^*^	46 (12.2)
≥90%^**^	153 (40.5)
**Key performance measure:** Complications after colonoscopy.	
**Question:** Percentage of patients in which immediate and delayed complications occurring after colonoscopy are monitored^a^.	
<95%	50 (13.1)
≥95%	133 (34.9)
I do not monitor complications after colonscopy	198 (52)
**Key performance measure:** Patient's experience.	
**Question:** Percentage of colonoscopies in which patient's experience during and after colonscopy is measured using validated scales^c^.	
<90%	60 (16)
≥90%^*^	47 (12.4)
Patient experience is not measured	272 (71.6)

* *Minimum standard according to ESGE performance measures*.

** *Target standard according to ESGE performance measures*.

a *Missing data for 11 participants*.

b *Missing data for 15 participants*.

c *Missing data for 12 participants*.

### Predictors of Adherence to Quality Performance Measures

Multivariable logistic regression analyses showed that working in an academic hospital (OR 2.04, 95% CI:1.22–3.4) and an endoscopy practice duration of >10 years (OR 2.39, 95% CI: 1.18–4.82) were significant predictors of adherence to performance measures for upper GI endoscopy (Table 4). Attending training courses on the quality of endoscopy in the last 5 years was associated with better adherence to performance measures for both upper (OR 1.93, 95% CI: 0.94–3.94, *p* = 0.07) and lower (OR 1.96, 95% CI: 0.91–4.2, *p* = 0.08) GI endoscopy, with a borderline significance (Tables 4, 5). When compared with participants from Northern Italy, those from the center/south were less adherent to performance measures for both EGD (OR 0.40, 95% CI: 0.25–0.65) and colonoscopy (OR 0.50, 95% CI: 0.30–0.82) (Tables 4, 5).

**Table 4:** Predictors of adherence to performance measures for upper gastrointestinal endoscopy in at least 6 out of 8 questions (respondents *n* = 391).

**Variable**	**Adherence to upper GI** **performance measures** **in <6 questions** ***n* = 287**	**Adherence to upper GI** **performance measures** **in ≥6 questions** ***n* = 104**	
	***n* (%)**	***n* (%)**	**OR (95%CI)**
Age, years
<50	140 (48.8)	41 (39.4)	1
≥50	147 (51.2)	63 (60.6)	0.95 (0.51–1.76)
Gender
Female	96 (33.5)	41 (39.4)	1
Male	191 (66.5)	63 (60.6)	0.63 (0.38–1.06)
Area of residence
North	167 (58.2)	36 (34.6)	1
Center/South	120 (41.8)	68 (65.4)	0.40 (0.25–0.65)
Practice setting
Community/private hospital	222 (77.4)	66 (63.5)	1
Academic hospital	65 (22.6)	38 (36.5)	2.04 (1.22–3.4)
Endoscopy practice duration
<10 years	100 (34.8)	21 (20.2)	1
≥10 years	187 (65.2)	83 (79.8)	2.39 (1.18–4.82)
Attendance to training courses on quality of endoscopy in the last 5 years
No	57 (19.9)	11 (10.6)	1
Yes	230 (80.1)	93 (89.4)	1.93 (0.94–3.94)

*GI, gastrointestinal; OR, Odds ratio; CI, confidence interval*.

**Table 5:** Predictors of adherence to performance measures for lower gastrointestinal endoscopy in at least 5 out of 6 questions (respondents *n* = 381).

**Variable**	**Adherence to lower GI performance measures in <5 questions *n* = 294**	**Adherence to lower GI performance measures in ≥5 questions *n* = 87**	
	***n* (%)**	***n* (%)**	**OR (95%CI)**
Age, years
<50	144 (49)	32 (36.8)	1
≥50	150 (51)	55 (63.2)	1.04 (0.55–1.99)
Gender
Female	107 (36.4)	29 (33.3)	1
Male	187 (63.6)	58 (66.7)	0.97 (0.57–1.68)
Area of residence
North	165 (56.1)	33 (37.9)	1
Center/South	129 (43.9)	54 (62.1)	0.50 (0.3–0.82)
Practice setting
Community/private hospital	216 (73.5)	63 (72.4)	1
Academic hospital	78 (26.5)	24 (27.6)	1.05 (0.6–1.83)
Endoscopy practice duration
<10 years	101 (34.4)	17 (19.5)	1
≥10 years	193 (65.6)	70 (80.5)	1.90 (0.9–4)
Attendance to training courses on quality of endoscopy in the last 5 years
No	58 (19.7)	9 (10.3)	1
Yes	236 (80.3)	78 (89.7)	1.96 (0.91–4.2)

*GI, gastrointestinal; OR, Odds ratio; CI, confidence interval*.

## Discussion

Our study showed that the adherence of endoscopists to ESGE quality performance measures for upper and lower GI endoscopy is sub-optimal in Italy. Most endoscopists do not adhere to several performance measures for EGD and colonoscopy.

With regard to EGD, most endoscopists do not measure procedure duration, provide accurate photo documentation, monitor complications after a therapeutic procedure, and register patients with BE. The duration of EGD seems to be associated with the likelihood to detect precancerous lesions and cancer in the upper GI tract (10, 11). For example, an examination time of at least 7 min from intubation to extubation was associated with about 3-fold detection of dysplasia and gastric cancer (12). Unfortunately, in our survey, only a minority of endoscopists recorded the duration of EGD, possibly increasing the chance of missing esophageal and gastric dysplasia and malignancy. Similarly, only half of the participants referred to provide accurate photo documentation of the upper GI tract. Likewise, previous studies in Europe found that only 35–70% of endoscopic reports included adequate photo documentation (13, 14). This may hamper communication with other health care providers and appropriate care and follow-up of patients (15). The use of novel electronic reporting systems including obligatory fields for recording procedure duration and allowing to make photos easier and less time-consuming, should be strongly encouraged to improve adherence to such performance measures (16).

We found that only a minority of participants followed the ESGE recommendation to monitor complications after therapeutic EGD. Monitoring complications is essential to assess the safety of procedures and identify potential targets for improvement. For example, the perforation rate after Savary or pneumatic dilation should not exceed 1%, whereas perforation and bleeding after endoscopic mucosal resection should be within 2% and 10%, respectively (2). Modern reporting systems that allow recording immediate complications, coupled with reliable methods to identify delayed complications, such as direct contact with patients through a phone call or analysis of hospital records for re-admission, should be promptly adopted in the endoscopic units to implement this performance measure (2, 8).

Guidelines recommend recording all patients with BE in a registry for a better follow-up; this may improve adherence to surveillance recommendations and the monitoring of cancer incidence (2). Unfortunately, in our study, more than half of the participants did not have a BE registry at all, and only 17% complied with the minimum standard of this performance measure (i.e., 85% of patients with BE). This finding would partially explain the suboptimal adherence to BE surveillance guidelines in Italy (17). Reporting systems that allow an automatic linkage with other databases using unique patient identifiers should avoid double entry of data and should facilitate the implementation of Barrett's patient registries (8).

With regard to the quality of lower GI endoscopy, we found that a quarter of endoscopists have a suboptimal ADR of <25%, only half used an appropriate polypectomy technique, and a minority registered complications after colonoscopy and measured patient' experience during and after the procedure.

Adenoma detection rate is inversely associated with CRC incidence and mortality, which is higher when colonoscopy is performed by an endoscopist with an ADR of <25% (6, 18). In our survey, the majority of participants declared ADR of ≥25% in agreement with recent population-based studies (19); however, one-quarter of them did not meet this threshold. This suboptimal ADR was not due to a low cecal intubation rate, as almost all participants declared to reach the cecum in at least 95% of cases. Targeting ADR might be practical; in the UK, after a nationwide audit showed sub-optimal ADR, quality improvement initiatives yielded substantial improvements of this measure over time (20).

With regard to the management of pathology, appropriate polypectomy includes biopsy forceps removal of polyps of ≤3 mm and snare (cold or with diathermy) polypectomy for larger polyps (3). In our survey, only half of the participants used the snare to resect polyps of >3 mm in the ESGE minimum standard (i.e., 80% of polyps). Inappropriate polypectomy techniques may increase the risk of incomplete polyp removal and, consequently, interval CRCs.

As for upper GI endoscopy, we found that only a minority of participants monitored complications occurring after diagnostic or therapeutic colonoscopy. Our study provides further evidence that the measurement of post-colonoscopy complications has only partially entered routine practice, as only half of the endoscopic centers report post-colonoscopy complications in Europe (21, 22). In the Netherlands, where the Dutch national colonoscopy registry allows continuous monitoring of complications, post-colonoscopy complication rates are around 0.5%, which is the ESGE minimum standard (23).

Colonoscopy may be a painful and embarrassing procedure and this may negatively affect diagnostic work-up for abdominal symptoms, participation in screening programs, and adherence to surveillance recommendations (24, 25). Patient satisfaction is an increasingly recognized quality indicator in endoscopy; some evidence from colonoscopy studies shows that higher-quality procedures lead to better patient experience (26). Unfortunately, our survey showed that most endoscopists in Italy did not measure patient experience at all, while only 12.4% of participants met the minimum standard (i.e., 80% of cases). Our data are in line with a survey across European countries showing that only 25% of endoscopic centers recorded patient satisfaction after colonoscopy (21). This underperformance may be due to several factors including cultural issues, lack of standardized approach to monitor patient experience, and logistic limitations related to the collection of patient feedback.

We found that endoscopists from academic hospitals and those more experienced, i.e., endoscopists who practiced for more than 10 years, were more adherent to performance measures for upper GI endoscopy. These associations were not found for lower GI endoscopy, possibly due to the more recent development of quality measures for upper GI endoscopy. In our study, endoscopists who attended previous training courses on the quality of endoscopy were more likely to adhere to performance measures for both upper and lower GI endoscopy, although the association was of borderline significance. We previously reported that attending training courses on BE significantly improves adherence to BE guidelines (17), and our finding would further support the need to implement training courses on the quality of endoscopy. We also found that endoscopists from the south and center of Italy were less adherent to quality measures for both upper and lower GI endoscopy. Economic resources play a role in improving endoscopy quality, and the lower availability of resources in the south and center of Italy may partially explain this finding.

To our knowledge, this is the first survey on adherence of endoscopists to ESGE quality performance measures for upper and lower GI endoscopy. Three surveys carried out in Spain (27), Italy (28), and across Europe (21) assessed the quality of lower GI endoscopy but were based on the 2012 ESGE position statement including only some of the most recent colonoscopy quality criteria. Furthermore, our survey provided a comprehensive questionnaire including performance measures of almost all pre-, intra-, and post-procedure quality domains for both upper and lower GI endoscopy.

This study has several limitations. The main limitation is the low participation rate (40%), which is in line with the low response rate (31%) of a previous SIED survey on BE (17). In addition, we do not have data on the characteristics of non-respondents that may differ from those of respondents, introducing potential selection bias. In addition, our study sample was similar to the population of SIED members in terms of age and gender, but not for the area of residence and practice setting. Other limitations of this study are inherent to questionnaire-based surveys, which are prone to self-reporting bias with responses that may be skewed toward adherence to guidelines. Finally, there is a general delay from the publication of recommendations to their implementation in routine clinical practice (29), and our survey was only carried out after 1–2 years since the publication of ESGE guidelines.

In conclusion, our study showed that adherence of endoscopists to ESGE performance measures for upper and lower GI endoscopy is suboptimal in Italy. This is in line with data from the SIED accreditation project showing that many Italian endoscopy centers fail to meet important quality indicators (30). National scientific societies have a pivotal role in the dissemination and implementation of quality criteria for GI endoscopy by endorsing and adapting European performance measures (31). Our data would support the need to implement performance measures and endorse educational and scientific interventions on the quality of endoscopy in Italy. Quality measures should also be embedded in trainees' programs to obtain better adherence results.

## Data Availability Statement

The raw data supporting the conclusions of this article will be made available by the authors, without undue reservation.

## Ethics Statement

Ethical review and approval was not required for the study on human participants in accordance with the local legislation and institutional requirements. The patients/participants provided their written informed consent to participate in this study.

## Author Contributions

RZ, HB, SC, AM, and AT conceived the study and drafted the protocol. RZ, LeF, and LoF performed the statistical analysis and drafted the manuscript. All authors revised the manuscript and approved the final version.

## Conflict of Interest

AT is a consultant for Boston Scientific and Olympus. The remaining authors declare that the research was conducted in the absence of any commercial or financial relationships that could be construed as a potential conflict of interest.

## Publisher's Note

All claims expressed in this article are solely those of the authors and do not necessarily represent those of their affiliated organizations, or those of the publisher, the editors and the reviewers. Any product that may be evaluated in this article, or claim that may be made by its manufacturer, is not guaranteed or endorsed by the publisher.
