# Corrigendum: Adherence to European society of gastrointestinal endoscopy quality performance measures for upper and lower gastrointestinal endoscopy: a nationwide survey from the Italian society of digestive endoscopy

**DOI:** 10.3389/fmed.2024.1406746

**Published:** 2024-04-09

**Authors:** Rocco Maurizio Zagari, Leonardo Frazzoni, Lorenzo Fuccio, Helga Bertani, Stefano Francesco Crinò, Andrea Magarotto, Elton Dajti, Andrea Tringali, Paola Da Massa Carrara, Gianpaolo Cengia, Enrico Ciliberto, Rita Conigliaro, Bastianello Germanà, Antonietta Lamazza, Antonio Pisani, Giancarlo Spinzi, Maurizio Capelli, Franco Bazzoli, Luigi Pasquale

**Affiliations:** ^1^Gastroenterology Unit, IRCCS Azienda Ospedaliero-Universitaria di Bologna, Bologna, Italy; ^2^Department of Medical and Surgical Sciences, University of Bologna, Bologna, Italy; ^3^Gastroenterology and Endoscopy Unit, Azienda Ospedaliera-Universitaria Policlinico di Modena, Modena, Italy; ^4^Gastroenterology and Digestive Endoscopy Unit, Pancreas Institute, University of Verona, Verona, Italy; ^5^Diagnostic and Therapeutic Endoscopy Unit, Fondazione IRCCS Istituto Nazionale Tumori, Milan, Italy; ^6^Digestive Endoscopy Unit, Fondazione Policlinico Universitario A. Gemelli IRCCS, Università Cattolica del Sacro Cuore, Rome, Italy; ^7^Digestive Endoscopy Unit, S. Luca Hospital, Lucca, Italy; ^8^Digestive Endoscopy Unit, Manerbio Hospital, Manerbio, Italy; ^9^Gastroenterology and Digestive Endoscopy Unit, S. Giovanni di Dio Hospital, Crotone, Italy; ^10^Gastroenterology and Digestive Endoscopy Unit, Baggiovara University Hospital, Baggiovara, Italy; ^11^Gastroenterology and Digestive Endoscopy Unit, S. Martino Hospital, Belluno, Italy; ^12^Department of Surgery “Pietro Valdoni”, University La Sapienza, Rome, Italy; ^13^National Institute of Gastroenterology IRCCS Saverio de Bellis, Castellana Grotte, Bari, Italy; ^14^Gastroenterology and Endoscopy Department, Valduce Hospital, Como, Italy; ^15^Kiwa Cermet Certification Body, Statistical Department, Bologna, Italy; ^16^UOC Gastroenterologia ed Endoscopia Digestiva, Ospedale Frangipane, Avellino, Italy

**Keywords:** endoscopy, quality, performance measure, ESGE, guidelines

In the published article, there was an error in affiliation 13. Instead of “Gastroenterology and Digestive Endoscopy Unit, National Institute of Gastroenterology “Saverio de Bellis”, Research Hospital, Castellana Grotte, Bari, Italy,” it should be “National Institute of Gastroenterology IRCCS Saverio de Bellis, Castellana Grotte, Bari, Italy.”

The authors apologize for this error and state that this does not change the scientific conclusions of the article in any way. The original article has been updated.

