# Supplementary material for: Adherence to European Society of Gastrointestinal Endoscopy Quality Performance Measures for Upper and Lower Gastrointestinal Endoscopy: A Nationwide Survey From the Italian Society of Digestive Endoscopy
Source: Front Med (Lausanne). 2022 Apr 6;9:868449. doi: 10.3389/fmed.2022.868449 (PMC9018975; doi:10.3389/fmed.2022.868449)
Supplement: Supplementary file 1 [file Table_1.DOCX]

**Appendix 1.** Questionnaire.

**Section I - demographic and professional characteristics of participants.**

1. **Gender**

- Male
- Female

1. **Date of birth** _____/_______/_______
2. **Area of residence**

- North-West **(**Valle d’Aosta, Piedmont, Lombardy, Liguria)
- North-East (Friuli-Venezia Giulia, Veneto, Emilia Romagna, Trento/Bolzano)
- Center (Tuscany, Marche, Umbria, Lazio)
- South and Islands (Abruzzo, Molise, Campania, Apulia, Basilicata, Calabria, Sicily, Sardinia)

1. **In which hospital do you work?**

- Community hospital
- University hospital
- Private hospital

1. **How many years have you been practicing endoscopy?**

- <5
- 5-10
- 11-15
- 16-20
- >20

1. **Have you attended a course on quality of digestive endoscopy over the last 5 years?**

- No
- Yes

**Section II - Quality performance measures for upper GI endoscopy.**

1. **Which is the percentage of your endoscopy reports recording the duration of the procedure from intubation to extubation ?**

- I do not record the duration of the procedure
- ≤30%
- ≤60%
- <90%
- ≥90%

1. **Which is the percentage of your endoscopy reports with accurate photodocumentation of anatomical landmarks** (duodenum, major papilla, antrum, angulus, corpus, retroflex of the fundus, diaphragmatic indentation, upper end of the gastric folds, squamocolumnar junction, distal and proximal esophagus, i.e. at least 10 images in total) **and abnormal findings ?**

- I do not record the duration of the procedure
- ≤30%
- ≤60%
- <90%
- ≥90%

1. **Which classification do you use for Barrett’s esophagus?**

- Simple description of length of Barrett’s mucosa
- Short and Long Barrett’s esophagus
- Prague C & M classification
- None

1. **Which classification do you use for erosive esophagitis?**

- Hetzel-Dent classification (Grade 1-4)
- Savary-Miller classification (Grade 1-4)
- Los Angeles classification (Grade A-D)
- None

1. **Which biopsy protocol do you use for Barrett’s esophagus?**

- 1-2 random biopsies along the Barrett’s epithelium
- 3-4 random biopsies along the Barrett’s epithelium
- 4 biopsies taken every 2 cm along the Barrett’s epithelium (Seattle protocol)
- I do not take biopsies

1. **Which is the percentage of patients that you monitor for complications after therapeutic upper GI endoscopy?**

- ≤30%
- ≤60%
- <95%
- ≥95%
- No registry for complications after endoscopy

1. **Which biopsy protocol do you use for identification of gastric atrophy and intestinal metaplasia**?

- 1 biopsy from the antrum and 1 biopsy from the corpus
- ≥2 biopsies only from the antrum
- ≥2 biopsies from the antrum and ≥2 from the corpus
- None

1. **Which is the percentage of patients with BE that you enter into a registry to monitor the incidence of dysplasia?**

- ≤30%
- ≤60%
- <85%
- ≥85%
- I do not have a registry for Barrett’s esophagus

**Section III - Quality performance measures for lower GI endoscopy**.

1. **Which is the bowel preparation scale that you use for measuring bowel preparation quality?**

- Boston Bowel Preparation Scale (BBPS)
- Ottawa Scale
- Aronchick Scale
- None

1. **Which is the percentage of screening or diagnostic colonoscopies in which you reach and visualize the whole cecum and its landmarks?**

- <80%
- ≥80%
- ≥90%
- ≥95%

1. **Which is the percentage of colonoscopies in patients aged 50 years or older in which you identify at least one adenoma?**

- ≤10%
- ≤15%
- <25%
- ≥25%

1. **Which is the percentage of polyps >3 mm in size that you remove with snare polypectomy (cold or with diathermy)?**

- <70%
- ≥70%
- ≥80%
- ≥90%

1. **Which is the percentage of patients in which you register immediate (perforation, bleeding or sedation-related adverse events) and delayed (7- or 30-day readmission rate and 30-day mortality rate) complications occurring after screening, diagnostic, or therapeutic colonoscopy?**

- ≤30%
- ≤60%
- <90%
- >90%
- I do not register complications

1. **Which is the percentage of colonoscopies in which patient experience during and after the procedure is measured using validated scales (i.e. the Global Rating Scale, the Gastronet, or others)?**

- ≤30%
- ≤60%
- <90%
- >90%
- I do not measure patient experience
